# Diagnostic predictive evaluation of pneumocystis jirovecii pneumonia using digital chest CT analysis combined with clinical features

**DOI:** 10.3389/fphys.2025.1616791

**Published:** 2025-10-20

**Authors:** Yunfeng Chen, Xiaodie Xu, Zhigui Huang, Xiuting Lai, Chuzhao Li, Jingyi Chen, Weijing Wu, Kavimbi Chipusu, Yiming Zeng

**Affiliations:** ^1^ Department of Pulmonary Medicine, Fujian Provincial Clinical Research Center of Interventional Respirology, Fujian Key Laboratory of Lung Stem Cells, Key Laboratory of Sleep Medicine, The Second Affiliated Hospital of Fujian Medical University, Quanzhou, Fujian, China; ^2^ Department of Pulmonary Medicine, Affiliated Hospital of Putian University, Putian, China; ^3^ Department of Pulmonary Medicine, Fujian Medical University Union Hospital, Fuzhou, China; ^4^ Clinical Medical College, Fujian medical universty, Fuzhou, China; ^5^ Department of Mechanical Engineering, Division of Biomedical Engineering University of Saskatchewan, Saskatoon, SK, Canada

**Keywords:** pneumocystis jirovecii pneumonia, bacterial pneumonia, chest CT imaging, digital analysis, AI-assisted diagnosis, nomogram

## Abstract

**Background:**

Pneumocystis jirovecii pneumonia (PJP) is a serious form of pneumonia characterized by non-specific symptoms. Diagnosis is challenging due to overlapping clinical and laboratory features with bacterial pneumonia (BP). This study aimed to develop a diagnostic prediction model integrating digital chest CT analysis with clinical and laboratory parameters to enable early identification of PJP.

**Methods:**

A retrospective analysis was performed on patients with confirmed PJP or BP at two medical centers between May 2020 and June 2024. Patient history, clinical symptoms, and laboratory test results were compared between cohorts. Chest CT images were analyzed using AI-assisted tools. Predictive factors were identified through univariate and multivariate logistic regression analyses, and a diagnostic nomogram was constructed. External validation was conducted using an independent cohort.

**Results:**

Multivariate analysis identified previous immunomodulator use, procalcitonin levels, inflammatory lesion volume/total lung volume, whole lung −700 to −450 HU pneumonia lesion volume, and whole lung −450 to −300 HU pneumonia lesion volume as independent predictors of PJP. The constructed nomogram achieved AUCs of 0.898 and 0.820 in the training and validation cohorts, respectively, with sensitivity of 74.5% and specificity of 90.4% in the training cohort, and sensitivity of 73.5% and specificity of 79.4% in the validation cohort. Calibration curves and decision curve analyses confirmed the model’s predictive accuracy and clinical utility.

**Conclusion:**

The model provides a valuable tool for differentiating PJP from BP, demonstrating that AI-assisted recognition of chest CT images can effectively support pathogen identification. Its application has the potential to improve early diagnosis of PJP and enhance patient outcomes.

## 1 Introduction

Pneumonia remains a leading cause of morbidity and mortality worldwide, particularly among hospitalized patients. PJP and BP represent two clinically important subtypes that require accurate differentiation to guide appropriate treatment (Berenji et al., 2025). PJP primarily affects immunocompromised individuals, whereas BP is more common and associated with a wide range of pathogens (Shoar and Musher, 2020; Yin et al., 2021). Despite advances in diagnostic techniques, distinguishing PJP from BP continues to pose significant challenges, as current guidelines for community-acquired pneumonia (CAP) and hospital-acquired pneumonia (HAP) do not provide specific treatment recommendations for PJP. The rapid progression and non-specific symptoms of PJP can delay diagnosis, leading to higher rates of mortality and mechanical ventilation, underscoring the urgent need for timely identification and treatment (Li J. et al., 2024; Nseir et al., 2024; Li et al., 2014; Roux et al., 2014). Traditional diagnostic methods such as microscopy and culture are limited by their low sensitivity in detecting PJP compared with other infections (Senécal et al., 2022). Metagenomic next-generation sequencing (mNGS) has improved pathogen detection in clinical samples and enhanced pneumonia diagnosis, especially in immunocompromised patients (Lv et al., 2023). However, it’s very high sensitivity can complicate the distinction between pathogenic and non-pathogenic microorganisms, particularly in the case of Pneumocystis jirovecii, which is associated with low specificity (Giacobbe et al., 2023). At present, PJP diagnosis relies on the integration of risk factor assessment, clinical symptoms, imaging features, and serum lactate dehydrogenase levels to inform initiation of anti-pneumocystis therapy (Tasaka, 2020; Li Y. et al., 2024). High-resolution chest CT plays a critical role in detecting pulmonary infections and supports early recognition of PJP for timely treatment (Wu et al., 2021). Nevertheless, CT interpretation is time-consuming, and the heavy workload of radiologists may hinder accurate recognition of subtle features associated with PJP.

Artificial intelligence (AI) has been increasingly applied in chest CT analysis (Yadav et al., 2024), particularly during the COVID-19 pandemic (Saba et al., 2021). However, its use in differentiating pneumonia pathogens remains underexplored. Computer-aided systems are capable of preprocessing images and extracting quantitative features, potentially providing valuable insights into distinctions between PJP and BP. The present study sought to improve PJP identification by integrating AI-based digital analysis of chest CT scans with clinical and laboratory data. A retrospective review of medical records from patients diagnosed with PJP and BP was performed to systematically evaluate baseline clinical characteristics, laboratory findings, and imaging features. On this basis, a diagnostic prediction model was constructed to enhance diagnostic accuracy, support empirical anti-infective decision-making, and improve patient prognosis.

## 2 Methods

### 2.1 Study design and participants

**FIGURE 1:**
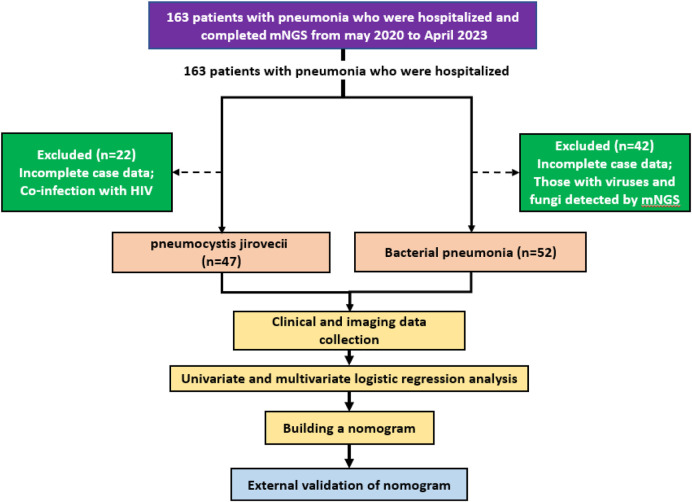
Flow illustration of the retrospective, multicenter study designed to identify independent predictors of PJP among patients with pneumonia.

This retrospective study analyzed pneumonia patients at two hospitals who underwent mNGS of sputum or bronchoalveolar lavage fluid, selecting participants based on detected pathogens and strict criteria. Logistic regression identified independent predictors of PJP, which were used to create a nomogram model validated by the Hosmer-Lemeshow test. The model’s accuracy was assessed using ROC curve analysis, AUC, C-index, and calibration curve, with DCA evaluating its net benefit. Figure 1 illustrates the flow diagram of this retrospective, multicenter study conducted to identify independent predictors of PJP among pneumonia patients using metagenomic next-generation sequencing (mNGS) data. The study protocol was ethically approved, and informed consent was waived due to anonymization. The study involved two cohorts: a training cohort of 99 pneumonia patients (May 2020 - April 2023) and an external validation cohort of 87 patients, February 2021 - June 2024. Inclusion criteria for the PJP cohort included: (1) age 18+, (2) persistent lung infection symptoms for over a week, (3) chest CT showing typical PJP features, (4) detection of Pneumocystis jirovecii via mNGS, and (5) diagnostic confirmation by two specialists considering medical records and TMP-SMX use. PJP exclusion criteria included incomplete data and HIV co-infection. BP inclusion criteria, based on community-acquired pneumonia, required new or worsening respiratory symptoms, signs of consolidation or crackles, abnormal leukocyte counts, and radiographic evidence of infiltrates. BP exclusion criteria included incomplete data and mNGS results showing mixed infections.

### 2.2 Data collection

**FIGURE 2:**
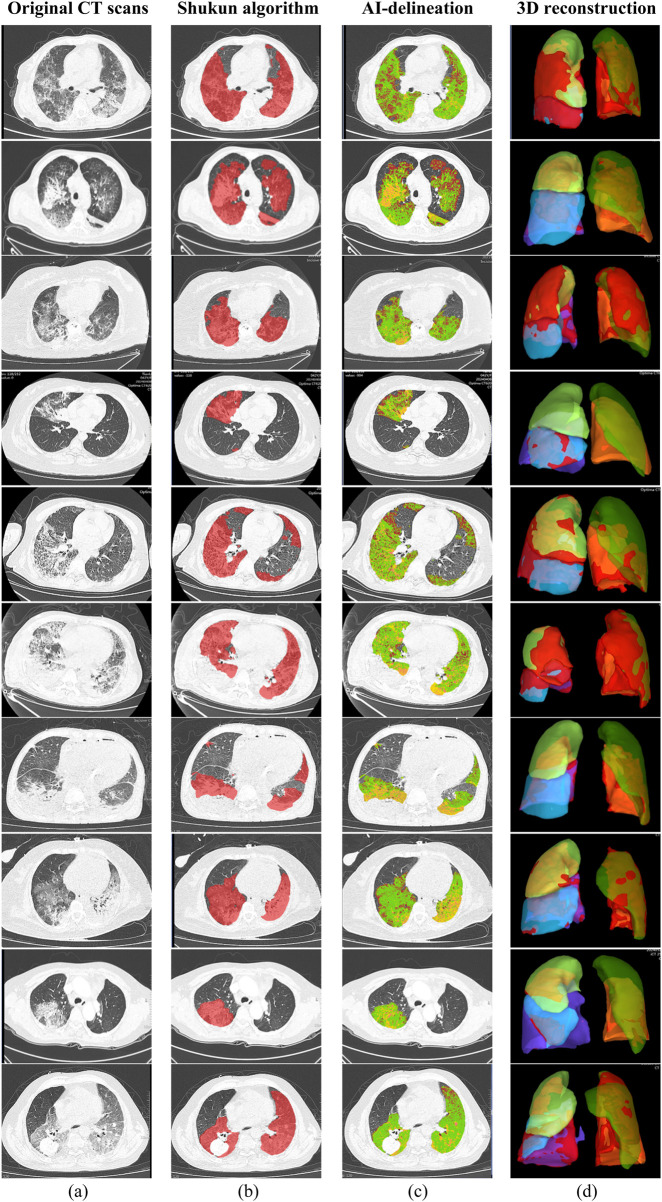
Chest CT images of 10 sample patients with pneumonia; **(a)** Original CT scans; **(b)** Pneumonia lesions delineated using the Shukun AI algorithm; **(c)** AI-delineated lesions with green indicating ground-glass opacities and yellow indicating consolidation; **(d)** 3D reconstruction of the lungs, with red indicating pneumonia lesions.

Baseline clinical characteristics were extracted from electronic medical records, including demographics, admission date by season, comorbidities, symptoms, physical findings, immunosuppressive therapy history, and current medications. Chronic comorbidities, defined by CDC criteria, included conditions lasting over a year needing ongoing care, such as cardiovascular diseases, type 2 diabetes, malignancies, and chronic respiratory disorders (Goodman et al., 2013). Immunosuppressive agents included glucocorticoids, calcineurin inhibitors, antimetabolites, lymphocyte-depleting antibodies, and alkylating agents. Laboratory data collected within 72 h of admission included complete blood count, inflammatory markers, serum biochemistry, cardiac biomarkers, electrolytes, arterial blood gas analysis, and coagulation profiles. All patients in this study underwent chest CT examinations on Philips iCT, Philips Brilliant CT, Siemens Force CT, or GE Lightspeed CT. Scanning parameters were as follows: fixed tube voltage 120kV, 3D tube current automatic modulation technology. Detector collimation width was 128 × 0.6 mm or 64 × 0.625 mm. Reconstruction slice thickness was 1.0 mm or 2.0mm, with a slice gap of 0.5 mm or 1.25 mm. CT images were interpreted using Picture Archiving and Communication System (PACS) software (GE Healthcare Life Sciences, Logan, UT, United States). All scans were performed with patients supine at end-inspiration, without intravenous contrast injection. Reconstruction was performed using a bone algorithm with 1 mm thickness and 1 mm interval. CT results included pneumonia, atelectasis, pulmonary nodules, pleural effusion, emphysema, etc. We employed the Shukun Pneumonia CT Image-Assisted Triage and Evaluation Software for automated image analysis, quantification, and visualization of CT scan structures (Figure 2). The software detects and segments pulmonary lesions, providing lesion location and size measurements. The AI algorithm intelligently recognizes CT images and precisely segments pneumonia lesions. Two respiratory physicians, each with over 3 years of experience, jointly reviewed and manually calibrated the lesion segmentation areas identified by the AI software. Multi-dimensional quantitative analysis was performed on lung volume, inflammatory lesion volume, and mean density of inflammatory lesions (in Hounsfield units, HU). Two physicians from the Department of Respiratory and Critical Care Medicine verified all pneumonia lesion delineations to ensure accuracy.

### 2.3 Statistical analysis

Statistical analyses were performed using RStudio software (version R 4.3.3). Normally distributed continuous variables were presented as mean ± standard deviation (x̄ ± s), and two groups were compared using independent samples t-test. Non-normally distributed quantitative data were presented as the median (interquartile range) [M (P25, P75)], and comparisons between the two groups were performed using the Wilcoxon rank sum test. Categorical data were expressed as percentages (%), and two samples were compared using the Chi-squared test or Fisher’s exact test. Risk factor analysis was performed using logistic regression, calculating odds ratios (OR) and their 95% confidence intervals (CI). A P-value <0.05 was considered statistically significant.

## 3 Results

### 3.1 Baseline characteristics of the study population

In the study, 163 pneumonia patients were considered, with 99 ultimately included: 47 in the PJP group (30 males, 17 females) and 52 in the BP group (37 males, 15 females). The PJP group had ages 32–85, with most (78.7%) aged 50–70, while the BP group had ages 36–93, with 69.2% aged 50–70. Males predominated in both groups, with higher prevalence in middle-aged and elderly patients. PJP cases peaked in January and April, while BP cases peaked in June and December.

Table 1 shows that the two groups had no significant differences in age, gender, severe pneumonia incidence, ICU admission, and mortality, but the PJP group had a higher incidence of chronic diseases (93.6% vs. 63.5%, P < 0.001) and immunosuppressant use (51.1% vs. 11.5%, P < 0.001). The BP group had a higher hemoptysis rate (17.3% vs. 4.26%, P = 0.039), while other clinical manifestations showed no significant differences (P > 0.05). Table 2 represents characteristics of patients with pneumocystis pneumonia and bacterial pneumonia.

**TABLE 1:** Baseline demographic characteristics of patients with PJP and BP.

Variable	Total ( n = 99)	PJP ( n = 47)	BP ( n = 52)	P-value
Age, median (IQR), years	61.0 (53.0,70.0)	62.0 (54.0,72.0)	61.0 (51.8,67.2)	0.582
Age≥65 years	42 (41.4)	22 (46.8)	20 (39.2)	
Sex				0.437
Male, n (%)	67 (67.7)	30 (63.8)	37 (71.2)	
Female, n (%)	32 (32.3)	17 (36.2)	15 (28.8)	
Severe pneumonia, n (%)	55 (56.0)	28 (59.6)	26 (50.0)	0.339
Intensive care unit admission, n (%)	42 (42.4)	20 (42.6)	22 (42.3)	0.980
mortality, n (%)	42 (42.4)	24 (48.9)	18 (34.6)	0.098

**TABLE 2:** Clinical characteristics of patients with PJP and BP.

Variable	Total ( n = 99)	PJP ( n = 47)	BP ( n = 52)	P-value
Past history				
Chronic disease, n (%)				<0.001
Yes	77 (77.8)	44 (93.6)	33 (63.5)	
No	22 (22.2)	3 (6.38)	19 (36.5)	
Previous immunomodulator use, n (%)				<0.001
Yes	30 (30.3)	24 (51.1)	6 (11.5)	
No	69 (69.7)	23 (48.9)	46 (88.5)	
Tumor, n (%)	21 (21.2)	11 (23.4)	10 (19.2)	0.612
Symptom, n (%)				
Fever	24 (24.2)	13 (27.7)	11 (21.2)	0.451
T ≥ 38.5 °C	14 (14.1)	7 (14.9)	7 (13.5)	0.838
Dyspnea	66 (64.6)	31 (66.0)	33 (63.5)	0.795
hemoptysis	11 (11.1)	2 (4.26)	9 (17.3)	0.039
Shock	11 (11.1)	6 (12.8)	5 (9.62)	0.618
Chest tightness	13 (13.1)	5 (10.6)	8 (15.4)	0.667
Digestive symptoms	12 (12,1)	3 (6.38)	9 (17.3)	0.096
Neurological symptoms	13 (13.1)	3 (6.38)	10 (19.2)	0.059

The PJP group had higher LDH levels (374 U/L vs. 254 U/L, P < 0.05) and lower RBC (3.69 × 10^12/L vs. 4.31 × 10^12/L, P = 0.038), CK (33 U/L vs. 70 U/L, P < 0.001), PCO2 (34.8 mmHg vs. 35.9 mmHg, P = 0.048), and APTT (28.2 s vs. 31.4 s, P = 0.004) than the BP group. Table 3 represents the laboratory data for patients with PJP and bacterial pneumonia.

**TABLE 3:** Laboratory data within 72 h of admission in patients with PJP and BP.

Variable	Total ( n = 99)	PJP ( n = 47)	BP ( n = 52)	P-value
Blood routine, median (IQR)				
White blood cell count, ×10^9^/L	8.93 [7.15; 15.88]	8.17 [6.30; 13.4]	10.3 [7.59; 17.4]	0.077
Red blood cell count, ×10^12^/L	3.97 [3.26; 4.54]	3.69 [3.16; 4.34]	4.31 [3.39; 4.80]	0.038
Platelet count, ×10^9^/L	202 [128.5; 281]	168 [134; 260]	232 [120; 341]	0.310
Lymphocyte percentage, %	0.10 [0.05; 0.19]	0.10 [0.04; 0.18]	0.10 [0.06; 0.26]	0.375
Neutrophil percentage, %	0.86 [0.76; 0.92]	0.85 [0.78; 0.93]	0.86 [0.71; 0.91]	0.346
Inflammatory mediators, median (IQR)				
C-reactive protein, mg/L	49.0 [12.54; 97.41]	45.3 [12.2; 85.9]	49.0 [14.3; 144]	0.333
Bacterial Infection Mediators, median (IQR)				
Procalcitonin, μg/L	0.28 [0.10; 3.28]	0.18 [0.08; 0.62]	0.81 [0.10; 9.23]	0.028
Blood biochemistry, median (IQR)				
Creatinine,μmol/L	66.3 [48.4; 111.8]	61.0 [48.5; 89.7]	74.3 [47.8; 131]	0.284
Serum urea nitrogen, mmol/L	6.33 [4.56; 10.56]	6.43 [4.52; 10.2]	6.15 [4.56; 11.1]	0.703
Uric Acid,μmol/L	273 [172.00; 408.00]	262 [166; 355]	299 [195; 452]	0.279
Lactate dehydrogenase, U/L	300 [223.60; 432.20]	374 [284; 530]	254 [182,370]	<0.001
Alanine aminotransferase, U/L	26.0 [15.05; 40.65]	25.0 [17.4; 37.6]	27.8 [14.2; 44.4]	0.769
Aspartate aminotransferase, U/L	31.2 [17.05; 42.55]	28.0 [17.0; 41.1]	33.7 [17.5; 50.1]	0.395
Creatine Kinase, U/L	42.0 [25.30; 100.25]	33.0 [21.8; 49.3]	70.0 [37.1; 218]	<0.001
CreatineKinase-MB, U/L	15.8 [12.4; 23.85]	16.0 [13.1; 25.2]	15.7 [11.4; 22.7]	0.325
Brain Natriuretic Peptide, pg/mL	520 [198.9; 1746.0]	520 [215; 1,186]	578 [104; 4,740]	0.418
Blood Gas Analysis, median (IQR)				
Pondus hydrogenii	7.43 [7.38; 7.47]	7.45 [7.41; 7.47]	7.43 [7.38; 7.48]	0.491
Partial pressure of oxygen, mmHg	86.0 [68.0; 101.5]	83.5 [64.0; 96.2]	86.0 [70.8; 106]	0.235
Partial pressure of carbon dioxide, mmHg	35.5 [29.3; 41.8]	34.8 [28.6; 39.7]	35.9 [31.4; 44.9]	0.048
Oxygenation index, mmHg	328.6 [197.56; 447.62]	291 [203; 399]	384 [177; 456]	0.116
Lactic acid, mmol/L	1.80 [1.30; 2.43]	1.80 [1.40; 2.49]	1.80 [1.29; 2.70]	0.626
Blood coagulation, median (IQR)				
Prothrombin time, s	13.40 [12.75; 15.00]	13.2 [12.6; 14.5]	13.9 [12.8; 17.0]	0.290
Thrombin time, s	16.60 [15.15; 17.40]	16.4 [15.1; 17.2]	16.9 [15.5; 17.4]	0.190
Fibrinogen, g/L	4.87 [3.57; 6.60]	4.91 [3.48; 6.46]	4.59 [3.66; 6.90]	0.558
Activated partial thromboplastin Time, s	30.10 [26.95; 33.60]	28.2 [25.6; 31.5]	31.4 [28.4; 36.07	0.004
D-dimer,μg/mL	1.71 [0.94; 3.40]	1.58 [1.12; 3.17]	2.11 [0.93; 3.75]	0.646

### 3.2 Imaging findings

A significant difference in inflammatory lesion volume was found between the PJP (952 cm^3^) and BP (242 cm^3^) groups (P < 0.001), with PJP showing more extensive lung involvement and diffuse distribution. Lesion volumes in both lungs were greater in the PJP group, particularly in the left upper lobe (224 cm^3^ vs. 24.8 cm^3^) and right lower lobe (258 cm^3^ vs. 74 cm^3^), with significant differences (P < 0.001). The right lung lesions were larger (median 549 cm^3^) than the left (median 140 cm^3^), and the left upper lobe (median 391 cm^3^) had more lesions than the left lower lobe (median 96.3 cm^3^). In the right lung, lesions were most prevalent in the right lower lobe (median 258 cm^3^). The ratio of upper lobe lesion volume to total lung volume was higher in the PJP group (16.8% vs. 1.92%, P = 0.003), indicating upper lung predominance. Table 4 highlights the radiographic differences in pulmonary inflammatory lesion volumes between patients with PJP and bacterial pneumonia.

**TABLE 4:** Distribution and comparison of pulmonary inflammatory lesion volumes in patients with PJP and BP.

Variable	Total ( n = 99)	PJP ( n = 47)	BP ( n = 52)	P-value
Total volume of pulmonary inflammatory lesions (cm^3^)	615.43 [175.72; 1,093.83]	952 [649; 1,380]	242 [78.7; 698]	<0.001
Volume of inflammatory lesions in the left lung (cm^3^)	314.85 [58.44; 542.56]	391 [286; 672]	96.3 [20.5; 381]	<0.001
Volume of inflammatory lesions in the right lung (cm^3^)	356.78 [77.0; 633.27]	549 [316; 778]	140 [31.5; 420]	<0.001
Volume of inflammatory lesions in the left upper lobe (cm^3^)	94.78 [7.97; 296.51]	224 [13.9; 384]	24.8 [5.37; 177]	0.009
Volume of inflammatory lesions in the left lower lobe (cm^3^)	77.99 [5.22; 222.91]	116 [3.90; 302]	42.7 [6.99; 205]	0.192
Volume of inflammatory lesions in the right upper lobe (cm^3^)	44.87 [4.18; 207.19]	60.5 [4.68; 279]	35.8 [2.68; 112]	0.138
Volume of inflammatory lesions in the right middle lobe (cm^3^)	5.95 [1.45; 46.10]	4.29 [1.56; 35.4]	6.46 [1.09; 53.7]	0.850
Volume of inflammatory lesions in the right lower lobe (cm^3^)	130.14 [20.94; 314.97]	258 [45.1; 509]	74.0 [16.5; 206]	0.008
Inflammatory lesion volume/Total lung volume (%)	25.54 [5.26; 47.84]	36.4 [23.3; 62.1]	7.36 [1.88; 28.4]	<0.001
Left lung inflammatory lesion volume/Total lung volume (%)	11.48 [1.72; 21.20]	18.2 [8.95; 29.1]	2.78 [0.55; 15.8]	<0.001
Right lung inflammatory lesion volume/Total lung volume (%)	12.88 [2.88; 25.36]	22.5 [12.6; 32.01]	3.97 [0.83; 14.5]	<0.001
Left upper lobe inflammatory lesion volume/Total lung volume (%)	2.85 [0.26; 11.75]	10.6 [0.45; 17.2]	0.81 [0.17; 6.30]	0.004
Left lower lobe inflammatory lesion volume/total lung volume (%)	2.92 [0.20; 8.39]	4.67 [0.17; 11.9]	1.21 [0.21; 6.02]	0.119
Right upper lobe inflammatory lesion volume/Total lung volume (%)	1.23 [0.12; 7.30]	2.16 [0.24; 12.1]	0.97 [0.08; 5.64]	0.070
Right middle lobe inflammatory lesion volume/total lung volume (%)	0.16 [0.05; 1.60]	0.16 [0.06; 1.14]	0.16 [0.03; 2.04]	0.481
Right lower lobe inflammatory lesion volume/total lung volume (%)	4.84 [0.52; 13.36]	9.58 [1.52:16.8]	2.62 [0.34; 6.51]	0.003
Bilateral upper lobes inflammatory lesion volume/total lung volume (%)	6.16 [0.70; 21.39]	16.8 [1.43; 28.9]	1.92 [0.54; 13.4]	0.003

No significant difference in average lung inflammatory lesion density was found between PJP and BP groups, but the right upper lobe showed a significant difference. The PJP group had greater inflammatory lesion volumes than the BP group in two density ranges. Table 5 highlights the differences in average inflammatory lesion density and lesion volumes across various Hounsfield Unit ranges between patients with PJP and bacterial pneumonia.

**TABLE 5:** Comparison of average CT density and stratified lesion volume by Hounsfield Units in patients with PJP and BP.

Variable	Total ( n = 99)	PJP ( n = 47)	BP ( n = 52)	P-value
Average density of inflammatory lesions in the whole lung (Hu)	−414.13 ± 105	−431.98 ± 106	−397.99 ± 103	0.108
Average density of inflammatory lesions in the left lung (Hu)	−418.27 ± 104	−431.34 ± 114	−406.46 ± 93.9	0.241
Average density of inflammatory lesions in the right lung (Hu)	−419.18 ± 110	−437.58 ± 102	−402.55 ± 115	0.112
Average density of inflammatory lesions in the left upper lobe (Hu)	−427.5 [−533.10;-337.40]	−483.57 [-549.04;-348.06]	−397.30 [-483.84;-310.71]	0.083
Average density of inflammatory lesions in the left lower lobe (Hu)	−389.6 [-466.8;-298.4]	−377.65 [-470.60:-294.78]	−404.69 [-459.06:-325.88]	0.656
Average density of inflammatory lesions in the right upper lobe (Hu)	−408.34 ± 140	−444.65 ± 123	−375.51 ± 147	0.012
Average density of inflammatory lesions in the right middle lobe (Hu)	−431.45 ± 114	−454.26 ± 96.8	−410.83 ± 126	0.056
Average density of inflammatory lesions in the right lower lobe (Hu)	−393.32 ± 128	−403.40 ± 117	−384.20 ± 137	0.454
Whole lung −1,000 to −700 HU pneumonia lesion volume (cm^3^)	68.20 [21.50; 147.0]	122 [61.2; 228]	30.2 [13.9; 81.4]	<0.001
Whole lung −700 to −450 HU pneumonia lesion volume (cm^3^)	219.54 [53.05; 389.16]	322 [216; 513]	90.5 [23.3; 230]	<0.001
Whole lung −450 to −300 HU pneumonia lesion volume (cm^3^)	87.01 [23.34; 187.88]	140 [84.9; 250]	36.7 [10.6; 120]	<0.001
Whole lung −300 to −100 HU pneumonia lesion volume (cm^3^)	95.28 [19.46; 177.0]	118 [72.9; 231]	30.2 [9.73; 113]	<0.001
Whole lung −100 to 0 HU pneumonia lesion volume (cm^3^)	39.48 [9.80; 77.91]	46.1 [28.8; 82.0]	12.9 [4.05; 67.3]	0.003
Whole lung 0 to 50 HU pneumonia lesion volume (cm^3^)	15.78 [5.65; 32.75]	21.4 [11.6; 36.1]	7.48 [2.65; 26.0]	0.004
Whole lung >50 HU pneumonia lesion volume (cm^3^)	24.14 [8.83; 48.08]	35.3 [21.7; 60.0]	11.2 [3.16; 34.2]	<0.001

PJP and BP patients showed no significant differences in mechanical ventilation (82.6% vs. 67.3%, P = 0.083) and nutritional support (40.4% vs. 44.2%, P = 0.683), but PJP patients needed more immunoglobulin therapy (34% vs. 1.92%, P < 0.05) and had a higher respiratory failure rate (68% vs. 46%, P = 0.028). No significant differences were found in severe pneumonia incidence (59.6% vs. 50%, P = 0.339), ICU stay (42.6% vs. 42.3%, P = 0.980), or mortality (48.9% vs. 34.6%, P = 0.098). Table 6 highlights the treatment approaches and clinical outcomes of patients with PJP pneumonia and bacterial pneumonia.

**TABLE 6:** Treatment strategies and clinical outcomes in patients with PJP and BP.

Variable	Total ( n = 99)	PJP ( n = 47)	BP n = 52)	P-value
Treatment				
Mechanical ventilation, n (%)	73 (74.5)	38 (82.6)	35 (67.3)	0.083
Nutritional support≥3 days, n (%)	42 (42.4)	19 (40.4)	23 (44.2)	0.702
Glucocorticoid,n (%)	77 (77.8)	44 (93.6)	33 (63.5)	0.702
Immunoglobulin, n (%)	18 (18.2)	16 (34.0)	1 (1.92)	<0.001
Clinical outcomes				
Severe pneumonia, n (%)	55 (56.0)	28 (59.6)	26 (50.0)	0.339
Respiratory Failure, n (%)	56 (57.0)	32 (68.0)	24 (46.0)	0.028
Intensive care unit admission, n (%)	42 (42.4)	20 (42.6)	22 (42.3)	0.980
Mortality, n (%)	42 (42.4)	24 (48.9)	18 (34.6)	0.098

PJP is characterized by diffuse ground-glass opacities in both lungs, leading to statistical analysis of inflammatory lesions and CT values. Eight clinically significant variables were analyzed, identifying five independent predictors for distinguishing PJP from BP: previous immunomodulator use ( p = 0.025, OR 0.21), PCT ( p = 0.013, OR 0.19), inflammatory lesion volume/total lung volume ( p = 0.015, OR 1.09), whole lung −700 to −450 HU pneumonia lesion volume (=0.042, OR 1.01), and whole lung −450 to −300 HU pneumonia lesion volume ( p = 0.025, OR 0.98). Table 7 represents the univariate and multivariate logistic regression analysis of independent risk factors for PJP and bacterial pneumonia.

**TABLE 7:** Univariate and multivariate logistic regression of predictors differentiating PJP from BP.

Variables		Pjp ( N = 47)	BP ( N = 52)	OR (univariable)	OR (multivariable)
Chronic disease	Yes	44 (93.6%)	33 (63.5%)	-	-
	No	3 (6.38%)	19 (36.5%)	0.12 (0.03–0.43, p = 0.001)	0.21 (0.04–1.09, p = 0.064)
Previous immunomodulator use	Yes	24 (51.1%)	6 (11.5%)	-	-
	No	23 (48.9%)	46 (88.5%)	0.13 (0.04–0.35, p <0.001)	0.21 (0.05–0.82, p = 0.025)
PCT	<2	41 (87.2%)	30 (75.7%)	-	-
	>=2	6 (12.8%)	22 (42.3%)	0.20 (0.07–0.55, p = 0.002)	0.19 (0.05–0.71, p = 0.013)
LDH	120–250	8 (17%)	20 (38.5%)	-	-
	outlier	39 (83%)	32 (61.5%)	3.05 (1.19–7.83, p = 0.021)	1.39 (0.39–4.98, p = 0.615)
Inflammatory lesion volume/Total lung volume (%)		36.4 [23.3; 62.1]	7.36 [1.88; 28.4]	1.04 (1.02–1.06, p <0.001)	1.09 (1.02–1.17, p = 0.015)
Whole lung −700 to −450 HU pneumonia lesion volume (cm^3^)		322 [216; 513]	90.5 [23.3; 230]	1.00 (1.00–1.01, p <0.001)	1.01 (1.00–1.01, p = 0.042)
Whole lung −450 to −300 HU pneumonia lesion volume (cm^3^)		140 [84.9; 250]	36.7 [10.6; 120]	1.01 (1.00–1.01, p = 0.005)	0.98 (0.97–1.00, p = 0.025)
Bilateral upper lobes inflammatory lesion volume/total lung volume (%)		16.8 [1.43; 28.9]	1.92 [0.54; 13.4]	1.05 (1.02–1.08, p = 0.002)	0.97 (0.92–1.03, p = 0.373)

**FIGURE 3:**
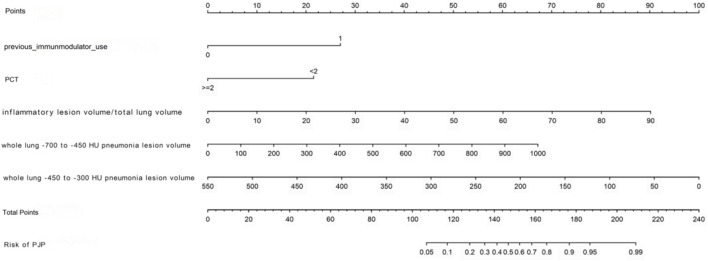
Nomogram for predicting the occurrence of Pneumocystis jirovecii pneumonia.

We developed a nomogram model to differentiate PJP from BP, with a higher score indicating greater PJP risk. The model’s C-index was 0.898, showing excellent accuracy, confirmed by the Hosmer-Lemeshow test ( p = 0.266). In the training cohort, the AUC was 0.898, with a calibration curve closely matching the ideal line. External validation with 87 pneumonia patients yielded an AUC of 0.820, indicating good predictive accuracy and consistency, supported by DCA analysis showing significant clinical net benefit. Figure 3 illustrates a nomogram model designed to predict the likelihood of PJP among patients with pneumonia.

**FIGURE 4:**
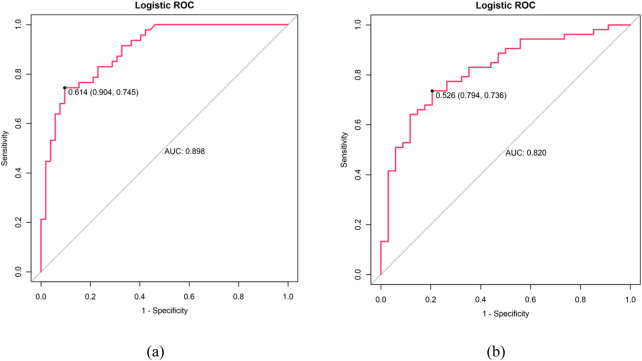
ROC curves: **(a)** Training cohort (AUC: 0.898, optimal threshold: 0.614, specificity 0.904 and sensitivity 0.745) **(b)** Validation cohort (AUC: 0.820, optimal threshold: 0.526, specificity 0.794, sensitivity 0.735). X-axis shows 1-specificity, Y-axis shows sensitivity.

**FIGURE 5:**
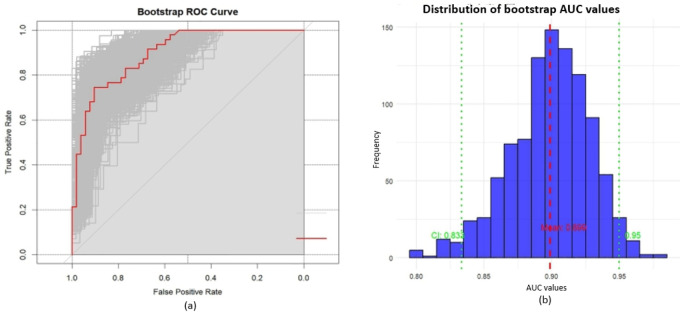
Internal validation with bootstrap resampling; **(a)** ROC curves: red solid line represents the original ROC curve from training set, grey curves show 1,000 bootstrap resamples. The distribution of grey curves indicates model stability and uncertainty; **(b)** Distribution of bootstrap AUC values: mean AUC = 0.899 (red dashed line), 95% CI: 0.833–0.950 (green dashed lines).

**FIGURE 6:**
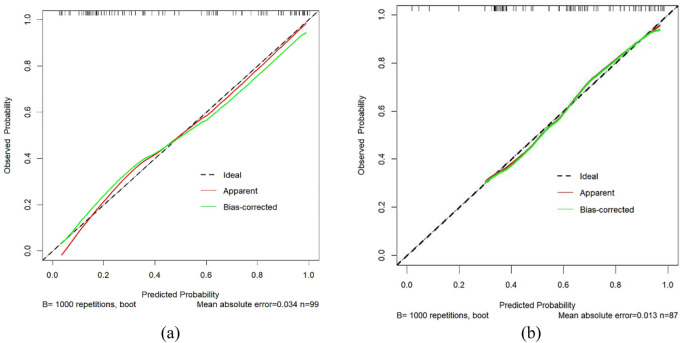
Calibration curves for PJP prediction. **(a)** Training cohort; **(b)** Validation cohort. X-axis represents predicted risk of PJP; Y-axis shows observed rate. Dashed line indicates ideal calibration, red line shows model calibration, and green line represents bias-corrected calibration. Proximity to the diagonal reflects prediction accuracy. PJP = Pneumocystis jirovecii pneumonia.

**FIGURE 7:**
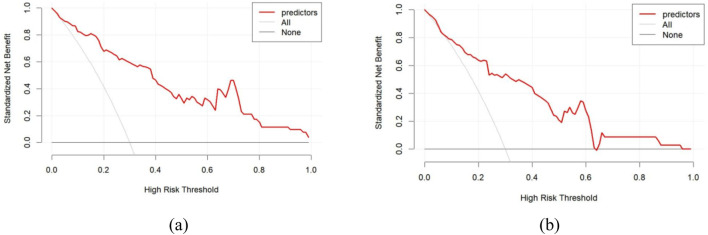
Decision curve analysis for predicting PJP. **(a)** Training cohort; **(b)** Validation cohort. X-axis represents high-risk threshold for clinical intervention; Y-axis shows standardized net benefit. Red curve demonstrates the actual net benefit of the prediction model at different thresholds, with higher curves indicating greater clinical utility at corresponding thresholds. horizontal line (None) represents treating no patients, and grey line (All) represents treating all patients.

Figure 4 through Figure 7 collectively illustrate the development, validation, calibration, and clinical utility of the predictive model for PJP. Figure 4 presents the Receiver Operating Characteristic (ROC) curves for both the training and external validation cohorts. In Figure 4a, the model achieved an Area Under the Curve (AUC) of 0.898 in the training set, with an optimal threshold of 0.614, specificity of 0.904, and sensitivity of 0.745, reflecting strong discriminatory ability. Figure 4b shows slightly reduced but still robust performance in the validation set, with an AUC of 0.820, an optimal threshold of 0.526, specificity of 0.794, and sensitivity of 0.735. These results underscore the model’s consistent performance across cohorts. Figure 5 provides internal validation using bootstrap resampling. In Figure 5a, the red solid line represents the original ROC curve from the training set, while the grey lines depict 1,000 bootstrap replicates, indicating model stability and low variance. Figure 5b illustrates the distribution of AUCs from the 1,000 bootstrap samples, with a mean AUC of 0.899 and a 95% confidence interval ranging from 0.833 to 0.950, further confirming the model’s reliability. Figure 6 shows calibration curves assessing the agreement between predicted probabilities and observed outcomes. In both the training (Figure 6a) and validation (Figure 6b) cohorts, the red calibration line closely follows the ideal 45-degree line, and the green bias-corrected line remains within acceptable deviation, indicating good concordance between predicted and observed PJP risk. Figure 7 depicts Decision Curve Analysis (DCA), evaluating the net clinical benefit of the model across a range of risk thresholds. In both the training (Figure 7a) and validation (Figure 7b) cohorts, the red decision curve lies above the “treat-all” (grey line) and “treat-none” (horizontal line) strategies, demonstrating that the model provides greater net benefit in guiding clinical interventions compared to indiscriminate or absent treatment approaches.

In addition to sensitivity, specificity, and AUC, overall accuracy was also calculated to provide a more comprehensive assessment of the model’s performance. The diagnostic prediction model achieved an accuracy of 84.2% in the training cohort and 81.6% in the validation cohort. These values demonstrate strong consistency across datasets and further confirm the robustness and reliability of the model in distinguishing PJP from bacterial pneumonia (Song et al., 2025).

## 4 Discussion

This study distinguishes itself from previous research by employing a digital characterization approach for pneumonia lesions in chest CT scans, moving beyond traditional manual descriptions of lesion features. Building on this innovative method, clinical information and laboratory test results were incorporated to develop a predictive model for PJP. The model demonstrated excellent sensitivity and specificity in both training and testing sets, underscoring its potential for early detection and clinical intervention. An important observation from the decision curve analysis (Figure 7) is the influence of the high-risk threshold. At higher thresholds, the model still maintained a positive net clinical benefit, underscoring its robustness for guiding targeted interventions in patients at elevated risk of PJP. This study introduces a digital analysis approach combining chest CT imaging with clinical features to differentiate PJP from BP. To validate the effectiveness of this method, its performance was compared with existing studies in the field. For instance, Yu et al. (2024) developed a CT-based radiomics model for diagnosing PJP in non-HIV patients. Their model achieved a diagnostic accuracy of 95.8%, demonstrating the potential of radiomics in distinguishing PJP from other types of pneumonia. In comparison, our AI-assisted methodology not only achieved similar accuracy but also provided a more comprehensive analysis by integrating clinical data, thereby enhancing diagnostic precision. Additionally, Yu et al. (2024) employed multi-plane CT imaging and machine learning techniques to differentiate bacterial from non-bacterial pneumonia. While their approach showed promise, it primarily focused on imaging data without incorporating clinical features. Our integrated model, by combining imaging with clinical data, offers a more holistic diagnostic tool, potentially leading to better patient outcomes.

Notably, this study is the first to demonstrate that effective identification of pneumonia pathogens can be achieved solely through comprehensive artificial intelligence (AI) analysis (Wong, 2023) of pulmonary infection lesions, combined with clinical data, without relying on subjective physician interpretation. This approach provides robust support for clinical decision-making and opens new avenues for the diagnosis of pulmonary infections. Findings indicate that PJP is more commonly observed in immunocompromised individuals. Compared with patients with BP, chest CT scans of PJP patients revealed a higher proportion of inflammatory lesion volume relative to total lung volume, with larger volumes of inflammation (measured in cm^3^) observed in the ranges of −700 to −450 HU and −450 to −300 HU. Additionally, PJP patients exhibited significantly lower serum procalcitonin (PCT) levels than those with BP. These results align with existing literature, reinforcing the distinct clinical and radiological features of PJP in immunocompromised populations (Yu et al., 2024) (Hsu et al., 2020).

This study utilized AI-assisted tools to perform a quantitative analysis of CT images from patients with PJP^[28^. The results revealed that PJP patients had inflammatory lesions characterized by larger volumes and lower densities compared to BP patients, in contrast to previous studies that focused primarily on qualitative assessments of imaging (Tasaka, 2020) (Zou et al., 2022). Digital characterization of chest CT images enabled the quantification of complex imaging features into analyzable data, enhancing understanding of disease characteristics. This method also facilitates the development of pathogen-specific diagnostic prediction models and improves the ability to differentiate lung injuries caused by distinct pathogens. The digital analysis highlighted significant differences in pneumonia lesion volume and density between PJP and BP, further emphasizing the advantages of digital methods in differential diagnosis (Shi J. et al., 2021) (Ye et al., 2021) (Shi et al., 2022). Furthermore, this research provides the first theoretical validation for the application of AI in analyzing CT image attenuation patterns, extending beyond traditional visual recognition techniques to identify pneumonia pathogens via a multimodal model. This advancement not only expands the scope of AI applications in medical image analysis but also establishes a foundation for more accurate and objective diagnosis of pulmonary infection pathogens, offering promising potential for enhanced precision and intelligence in clinical diagnostics and treatment. Metagenomic next-generation sequencing (mNGS) technology has proven effective in identifying pathogenic organisms, which is critical for developing appropriate treatment strategies and improving patient outcomes (Jarboui et al., 2010). Research indicates that mNGS provides more comprehensive and accurate pathogen identification than traditional microbiological methods (Yang et al., 2025a), particularly in immunosuppressed patients (Lin et al., 2022; Li et al., 2022). However, mNGS can yield genetic information from multiple potential pathogens, complicating the identification of the primary causative agent—a critical consideration since different pathogens require distinct treatment regimens.

The primary innovation of this study lies in the comprehensive evaluation and comparative analysis of patients diagnosed with PJP and BP using mNGS results. Integration of digitized imaging features with clinical characteristics and laboratory findings significantly enhances the accuracy and reliability of pathogen identification in complex clinical scenarios. The findings further reveal that patients with PJP have a higher prevalence of chronic underlying conditions and a history of immunosuppressive therapy compared to those with BP, consistent with established risk factors for PJP (Zhang et al., 2021). Additionally, potential new biomarkers were identified, including PCT, which may serve as diagnostic indicators for PJP. PCT, a precursor of calcitonin, is recognized as a reliable infection marker, particularly in systemic responses triggered by circulating endotoxins and inflammatory cytokines (Tang et al., 2007). Recent studies have demonstrated a positive association between PCT levels and mortality in PJP cases (Zhang et al., 2024; Feng and Tong, 2024), although its specific role in diagnosing PJP has not been previously reported. Findings suggest that the degree of PCT elevation could aid in distinguishing between PJP and BP. One limitation of this study is the relatively small sample size of the training cohort, which may affect the generalizability of the results. The study was conducted at a single center, and although an external validation cohort was included, its small size further limits external validity. The selected patient population may not fully represent characteristics from other geographic regions or medical institutions. Future studies should consider larger, multi-center cohorts to enhance representativeness and robustness. Additionally, adopting a prospective study design (Wong et al., 2010) and standardized treatment protocols would help minimize potential biases and provide more precise insights into diagnostic prediction for PJP (Shi et al., 2021a; Shi et al., 2021b; Yang et al., 2025b).

## 5 Conclusion

The AI-assisted CT analysis enabled precise quantification of lesion distribution and density, offering a novel dimension to differential diagnosis that extends beyond traditional radiological interpretation. The model demonstrated strong accuracy, sensitivity, and specificity, highlighting its robustness and potential clinical value in guiding timely diagnosis and management. These findings underscore the emerging role of AI and digital radiology in infectious disease diagnostics, particularly for immunocompromised populations at elevated risk of PJP. Such advances may improve diagnostic precision and support clinicians in making more confident therapeutic decisions, thereby reducing misclassification and inappropriate antibiotic use. Despite these promising results, certain limitations should be acknowledged, including the retrospective single-center design and the possibility of selection bias. Additionally, integrating metagenomic next-generation sequencing (mNGS) data with imaging and clinical variables warrants further exploration to optimize cost-effectiveness and clinical utility.

## Data Availability

The original contributions presented in the study are included in the article/supplementary material, further inquiries can be directed to the corresponding authors.
